# Cancer metabolism rewiring and chromatin methylation: a vulnerable epi-metabolic link

**DOI:** 10.3389/freae.2025.1638572

**Published:** 2025-08-25

**Authors:** Sandra Atlante, Veronica Barbi, Carlo Gaetano, Barbara Illi

**Affiliations:** ^1^ Institute for Systems Analysis and Computer Science, National Research Council (CNR) - IASI, Rome, Italy; ^2^ Istituti Clinici Scientifici Maugeri IRCCS, Pavia, Italy; ^3^ Institute of Molecular Biology and Pathology – National Research Council (IBPM-CNR), Rome, Italy

**Keywords:** oncometabolite, chromatin methylation, D-2 hydroxyglutarate, S-adenosine-L-methionine, ten-eleven translocation proteins, PRMT (protein arginine methyltransferase)

## Abstract

Over the past few years, significant advances have been made in understanding the crosstalk between cancer metabolism and gene expression. Whereas higher levels of expression of metabolic enzymes may be considered a conceivable compensatory mechanism to satisfy the increasing request of energy of tumor cells, the detection of changes in the amount and species of intermediate metabolites (oncometabolites) and the discovery of their functional role as co-factors and structural components of chromatin modifiers tightened the link between metabolic shifts and epigenome reshaping in cancer. Changes in the chromatin methylation landscape are one of the epigenetic fingerprints of cancer metabolic rewiring associated with the tumorigenic features of neoplasms. Thus, we propose targeting metabolic enzymes directly involved in cancer methylome remodeling and oncometabolite-dependent chromatin modifiers as innovative tools to reset the epigenome of deregulated cancer cells.

## Introduction

Metabolic pathway alterations are typical of several human diseases, including cancer. Type 2 diabetes and cardiometabolic syndrome are, probably, the best examples of the impact of dysmetabolic conditions on human health. In these pathological contexts, altered secretion of pro-inflammatory cytokines - adiponectin, leptin, resistin, plaminogen activator inhibitor - and also free fatty acids from accumulated adipose tissue negatively affects the insulin pathway, leading to insulin resistance, high plasma glucose levels and hyperinsulinemia (Fahed et al., 2022). Metabolic shifts have been also observed in other human pathologies, such as polycystic kidney (PKD) and Alzheimer’s diseases (AD). PKD, in the majority of cases, is a genetic autosomal dominant disease of the adult, caused by mutations in the *PKD1* (85%) and *PKD2* genes (10–15%) and characterized by the presence of slowly enlarging renal cysts. The kidney is exposed to high metabolic pressure, thus it is not surprising that PKD, beside its genetic background, is also the results of metabolic alterations which include enhanced glycolysis, altered lipid and aminoacids metabolism, increased autophagy and mitochondrial dysfunction (Nowak and Hopp, 2020). In AD brains and peripheral organs, mitochondrial dysfunction precedes dysmetabolism, due to decreased activity of enzymes belonging to both the tricarboxylic acid cycle (TCA) and the electron transport chain, this latter generating reactive oxygen species (ROS) from molecular O_2_, strongly contributing to AD pathology (Peng et al., 2020). Altered accumulation of metabolites has been reported first in peripheral tissues than in AD brains, with increased fatty acid β-oxidation and accumulation of acetyl-CoA (Peng et al., 2020). Notably, metabolic changes are also typical of non-pathological processes, as an adaptive mechanism to novel stimuli. This is the case of metabolic reprogramming in innate immune cells during the acquisition of trained immunity. In fact, upon microbial or endogenous ligands exposure, innate immune cells fuel glycolysis, the TCA cycle and the cholesterol pathway to satisfy their increased energy requirements (Domínguez-Andrés et al., 2020).

Metabolism derangement due to high energy demand represent a well-documented phenomenon occurring in cancer cells, especially when exposed to stress conditions, such as nutrient deprivation. The most well-known paradigm of cancer metabolism rewiring is aerobic glycolysis (Warburg et al., 1927), which, far from being a mitochondrial dysfunction, as initially hypothesized by Otto Warburg, is exploited by tumor cells to oxidize carbon units and produce ATP. Cancer metabolic reprogramming constantly refuels intracellular ATP amount and intermediates of the tricarboxylic acids cycle (TCA), which, in turn, serve to rapidly produce the macromolecules’ building blocks – amino acids, nucleotides, lipids, and fatty acids - essential to sustain fast cycling tumor cells (Li and Zhang, 2016). Specularly, macromolecules may be used to generate carbon units and intermediate metabolites, mostly derived from mitochondria. This bi-directional link relies on constant communication between mitochondria and the nucleus, known as the “mito-nuclear connection” (Walker and Moraes, 2022), and reflects the two-way communication between tumor metabolism and gene expression (Figure 1). Indeed, changes in metabolic fluxes within cancer cells induce the expression of key metabolic enzymes through the activation of transcription factors and co-regulators (Abdul-Wahed et al., 2017; DeBose-Boyd, and Ye, 2018), whereas metabolic intermediates have been demonstrated to serve as co-factors for chromatin remodellers and DNA modifiers (Campbell and Wellen, 2018). We are currently at the dawn of a new era, unlocking the deep connection between metabolic alterations and changes in the epigenomic landscape of cancer cells. A remarkable example is the detection in the nucleus of metabolic enzymes (Huangyang and Simon, 2018; He et al., 2017) which enables the proper production in space and time of intermediate metabolites, not-permeable to the nuclear membrane, such as Acetyl-CoA, the co-factor of acetyl-transferases - upon specific transcriptional requirements.

**FIGURE 1:**
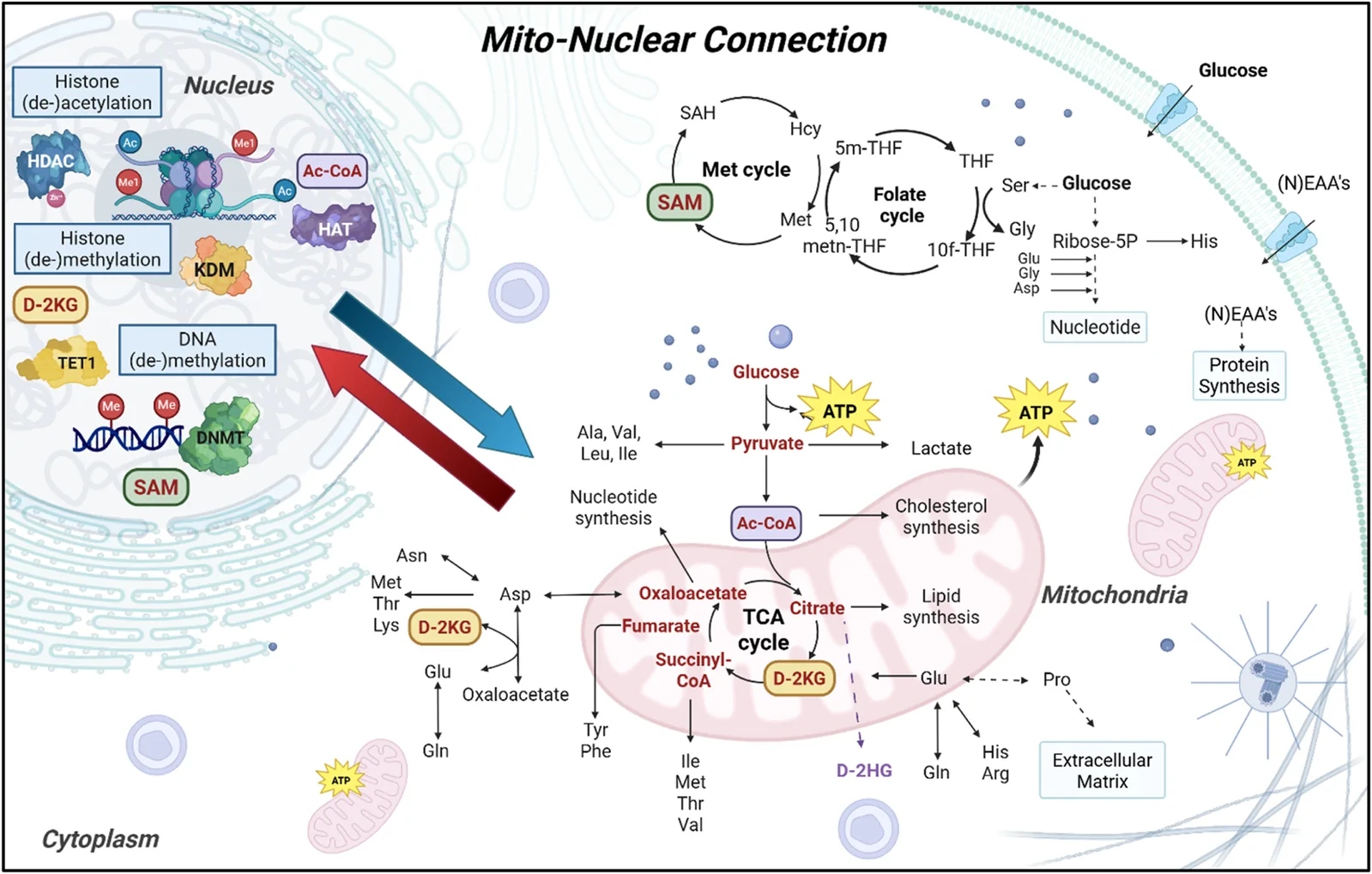
The Mito-Nuclear Connection. The cartoon shows the bidirectional interplay between mitochondria and the nucleus and highlights the reciprocal communication between tumor metabolism and gene expression. Cancer cells metabolism intermediates are channeled into the production of the macromolecular building blocks–amino acids, nucleotides, lipids, and fatty acids–essential for rapidly cycling cells, which, in turn, can be used to generate carbon units. Importantly, a variety of epigenetic enzymes exploit these metabolic intermediates as either substrates or co-factors, influencing gene expression through chromatin remodeling.

## Metabolism and chromatin methylation

Among the epigenetic modifications, DNA methylation is probably the most well-known. Generally associated with chromatin compaction and the repression of gene transcription, its role in gene activation has been reported, especially when methylated CpG islands are located within gene bodies (Lister et al., 2009). Chromatin methylating enzymes — both DNA (dnmts) and histone methyltransferases (HMTs) – require S-adenosylmethionine (SAM) as the donor of methyl groups. SAM may be synthesized by both the methionine cycle and a metabolic cross between the methionine and serine cycles, the latter regenerating methionine through the production of 5-methyl-tetrahydrofolate from glycine via the folate cycle. 5-methyl-tetrahydrofolate donates a methyl group to homocysteine, regenerating methionine (Locasale, 2013). Methionine adenosyl transferase (MAT) produces S-adenosylhomocysteine (SAH) when generating SAM (Locasale, 2013). SAM and SAH are permeable to the nuclear membrane, and chromatin is highly sensitive to the SAH/SAM ratio (Reid et al., 2017). Indeed, whereas SAM serves as a co-factor dnmts and HMTs, SAH has been demonstrated to be a methyltransferase inhibitor (Reid et al., 2017). Accordingly, methionine is essential for cancer cells; its deprivation results in decreased H3K4me3 levels in genomic regions encoding for genes involved in the execution of tumorigenic programs (Mentch et al., 2015; Dai et al., 2018) and in defective type I protein arginine methyltransferase 1 (PRMT1), whose activity is required to sustain the expression of the methionine transporter SLC43A2 via asymmetrical dimethylation of YAP. Furthermore, serine and/or methionine deprivation reduces genomic DNA methylation, with the consequent derepression of tumor suppressors (Białopiotrowicz et al., 2020).

As with any epigenetic modification, DNA methylation is reversible. Thus, DNA methylation implies also a DNA demethylation process. Disruption of the DNA methylation/demethylation cycle is one of the best examples of how metabolism deregulation may profoundly impact the cancer epigenome. In glioblastoma (GBM), the mutated isocitrate dehydrogenase (IDH) 1 leads to the accumulation of D-2-hydroxyglutarate (D-2HG) from D-2-ketoglutarate (D-2KG) (Chou et al., 2021). Mutated IDH1 is capable to use NADPH - produced during the generation of D-2KG from isocitrate — to reduce D-2KG into D-2HG (Du and Hu, 2021). This (onco)metabolite, in normal conditions, plays a role in immune cells’ biological activity. It inhibits lipopolysaccharides (LPS)-induced lactate production in dendritic cells (DCs), limiting interleukin-12 (IL-12) secretion and DCs activation, while increasing oxygen consumption (Ugele et al., 2019). Further, it regulates T lymphocytes differentiation. Indeed, in Th17 cells, glutamate oxalacetate transaminase 1 (GOT1) produces high levels of glutamine-derived D-2HG, leading to a hypermethylation of the *Foxp3* promoter and inhibiting Treg cells differentiation (Xu et al., 2017). An imbalance between Th17/Treg ratio has been found in asthma, where GOT1 activation promotes *Foxp3* silencing (Sun et al., 2020). Based on these evidences, it has been suggested that other autoimmune pathologies, characterized by Th17/Treg ratio imbalance, such as psorias and multiple sclerosis–may rely on D-2HG accumulation. Tumor-derived D-2HG, captured by T lymphocytes, limits respiration and ATP production, reducing T cells activity (Bunse et al., 2018), whereas in the heart, it induces a shift toward glycolysis, promoting cardiometabolic stress and contractile dysfunction (Karlstaedt et al., 2016).

In cancer cells, D-2HG negatively affects DNA demethylation. During this process, in normal metabolic conditions, D-2KG binds and activates the ten-to-eleven translocation (TET) family of enzymes (TET1, 2 and 3), which catalyze the oxidative deamination of 5-methylcytosine (5 mC) to 5-hydroxy-mC (5-hmC) and then to 5-formylC (5-fC) and 5-carboxyC (5-caC). The latters may be excised by thymine glycosylase (TDG), and the remaining abasic site repaired by the base excision repair (BER) enzymes, with the replacement of cytosine. On the contrary, D-2HG cannot bind TETs and impairs the proper accomplishment of the DNA demethylation cycle, promoting the occurrence of a hypermethylator phenotype (Noushmehr et al., 2010). The hypermethylator phenotype, besides altering CpG methylation pattern, has been also shown to negatively affect the proper organization of chromatin topologically associated domains (TADs; Xu et al., 2011), thus affecting the structural organization of the epigenome. Notably, D-2HG also inhibits histone lysine (K) demethylases (KDMs), thereby further strengthening chromatin compaction (Chowdhury et al., 2011). We have demonstrated that promoting D-2KG accumulation in breast cancer cells by inhibiting D-2KG dehydrogenase (KGDH), restores TETs activity, enhancing TET1, 2 and 3 expression, with a consequent increase in 5hmC (Atlante et al., 2018). Furthermore, KGDH inhibition modulated nitric oxide (NO) metabolism, inducing the increase of the microRNA 200 family members. This circuitry impaired the epithelial-mesenchymal transition (EMT) of breast cancer cells through the downregulation of the EMT factors Zeb1 and CtBP1. Indeed, D-2KG is a precursor of ornithine, which leads to the production of L-arginine, a substrate for NO synthases. This phenomenon is a notable example of a functional crosstalk among different metabolic cycles (TCA and arginine/nitric oxide metabolic cycles) and gene expression in cancer.

## Metabolism and chromatin modifiers

By definition, the term “chromatin modifier” includes all the enzymes involved in both DNA and histone modifications. In this regard, dnmts, TETs and any macromolecular complex able to modify histone tails, including HMTs, PRMTs and KDMs, all with methylating/demethylating activity, can be considered chromatin modifiers. The interaction properties of these molecules may be potently affected by the metabolic status within a given cell. Indeed, dysmetabolism-induced D-2HG impairs the formation of the TET1/TDG complex, pivotal for the DNA demethylation cycle and genome stability, leading to the cytosolic re-location of TET1. Most importantly, D-2HG overactivates TDG in an allosteric manner, enhancing its capacity to remove G/T mismatches and/or 5-fC (Spallotta et al., 2018). This phenomenon, first characterized in human cardiac mesenchymal stem cells (hCMSCs), has been also detected in pancreatic epithelial cells in which low D-2KG levels were induced by dysmetabolic conditions exposure. Intriguingly, in this system, type II PRMT5, which symmetrically dimethylates its targets (Blanc and Richard, 2017), was found to interact with TET1 and TDG. Upon D-2KG decrease, PRMT5 detached primarily from TDG, following TET1 in the cytosol (Illi B, original data). Symmetrical dimethylation of TDG was consistently detected under normal metabolic conditions and was lost upon exposure to dysmetabolism. Since the prominent effect of both symmetrical and asymmetrical dimethylation is the modification of protein interaction properties, it is conceivable that a dysmetabolic environment, affecting PRMT5 activity, promotes the loss of TDG symmetrical dimethylation and consequent detachment from TET1. Indeed, inhibiting PRMT5 in pancreatic epithelial cells with EPZ01566 – a PRMT5-specific inhibitor — exactly reproduced TET1/PRMT5/TDG complex disassembly (Illi B, original data). In parallel, an increase in asymmetrical dimethylated TDG species was detected (Illi B, original data). This effect was not surprising, as type I PRMT1 and type II PRMT5 share the same substrates, both histones and non-histone proteins (Favia et al., 2019); thus, it is conceivable that, in the absence of PRMT5, PRMT1 catalyzes TDG asymmetrical dimethylation. The functional effect of these modifications on TDG activity has to be still determined (Figure 2). However, these results further highlight the profound impact dysmetabolism has on chromatin modifiers, affecting the formation of remodelling complexes, which in turn lead to aberrant transcription. Importantly, in primary, IDH wild-type glioblastoma, PRMT1 is recruited onto 5hmC enriched chromatin domains by the chromatin target of protein arginine methyltransferase 1 (CHTOP), driving the asymmetrical dimethylation of arg 3 on histone H4 (H4R3me2as), allowing the ignition of transcriptional programs essential for glioblastomagenesis (Takai et al., 2014). Thus, loss of 5hmC in IDH-mutated GBMs due to the impairment of the DNA demethylation cycle as a consequence of D-2HG accumulation, is detrimental to GBM oncogenic properties. Indeed, secondary IDH-mutated GBMs have a better prognosis (Gargini et al., 2020). These results underly the heterogeneity of transcriptional responses of different cancer cells to metabolic cues and the need to face each specific tumor as a unique epi-metabolic entity. Of note, PRMT5 participates in the formation of the CHTOP/PRMT1 complex, although its function in this context has not been extensively studied (Takai et al., 2014). In our opinion, PRMT5 may serve as a platform granting the association between CHTOP and PRMT1, as occurs in mesenchymal glioblastoma stem cells (MesGSCs) for the Myc/PRTM1/PRMT5 ternary complex (Favia et al., 2019).

**FIGURE 2:**
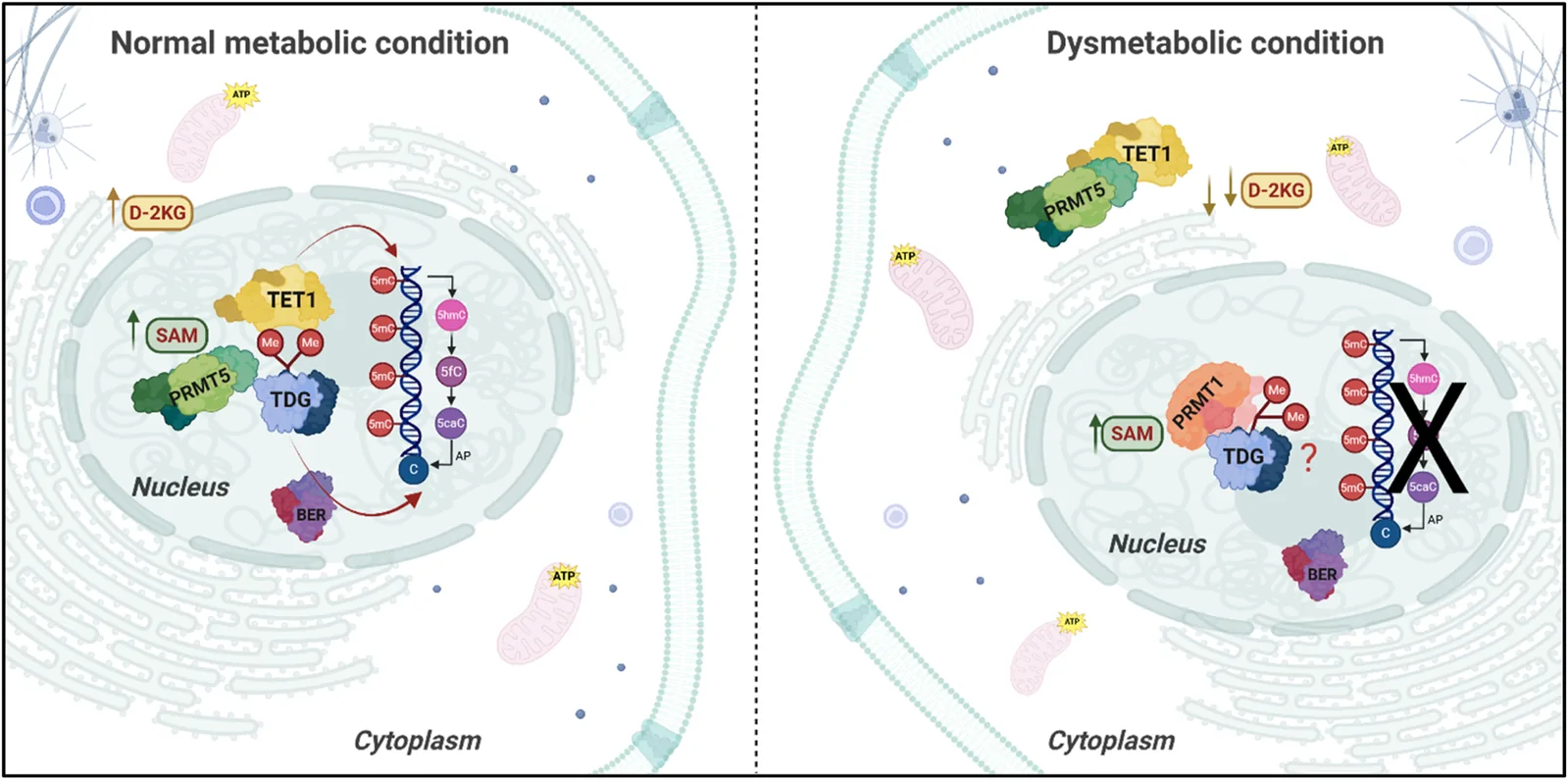
Impact of Dysmetabolic Conditions on DNA-demethylation and Repair Pathways. The picture illustrates the cellular localization and activity of key enzymes involved in DNA modification and repair depending on the cellular metabolic condition. Under normal metabolic conditions (left panel) D-2-ketoglutarate (D-2KG) levels are sufficient to support the demethylation activity of Ten-Eleven Translocation (TET) enzymes, while the Protein Arginine Methyltransferase 5 (PRMT5) interacts with TET1 protein and Thymine DNA Glycosylase (TDG) in the nucleus. Upon dysmetabolic conditions (right panel), characterized by decreased D-2KG levels, TET1 and PRMT5 relocate in the cytoplasm, leading to asymmetrical dimethylation of TDG and impairing the conversion of 5 mC to 5hmC, interfering with the demethylation cycle and potentially with the Base Excision Repair (BER) process, leading to unrepaired DNA damage, in the case of TDG overactivation.

## Discussion

Both R and K histone methyltransferases and dnmts are highly sensitive to the amount of SAM. Thus, it is not surprising that, to maintain chromatin and DNA hypermethylation, cancer cells enhance both the import and the production of amino acids like serine (McBride et al., 2024), which also allows methionine regeneration through a glycine-dependent shunt from the serine to the methionine cycle (Locasale, 2013). In parallel, the dysmetabolic environment characteristic of cancer cells may lead to decreased DNA demethylation via D-2HG-dependent TET activity impairment, TET/TDG complex disassembly, and TDG overactivation, highlighting a crosstalk between deregulated amino acid metabolism, the TCA cycle, and cancer cell methylome re-structuring. This strict relationship also reveals potential epi-metabolic vulnerabilities to be exploited for therapeutic purposes. Increasing the intracellular amount of D-2KG through KGDH inhibtion (Atlante et al., 2018) or blocking IDH mutants activity in IDH-mutated tumors (DiNardo et al., 2021), may represent a feasible strategy. In parallel, depleting SAM may impair cancer epigenome hypermethylation. SAM depletion may be accomplished by targeting the rate-limiting enzyme of the methionine salvage pathway, the methylthioadenosine phosphorylase (MTAP), whose loss have been demonstrated beneficial for diverse neoplasms (Affronti et al., 2020; Jing et al., 2020). Of note, the accumulation of MTAP substrate, methyltioadenosine, in MTAP-deficient cells sensitizes cancer to PRMT5 inhibition (Kryukov et al., 2016), further strenghtening the link between metabolism and this familiy of R-methyltransferases. The newly identified putative participation of PRMTs in the DNA methylation/demethylation cycle complex formation may suggest an intricate interaction between the TCA and the methionine/serine cycles. The decrease in D-2KG may not only results in TET1/TDG dissociation but also in PRMT5 detachment and loss of TDG symmetrical dimethylation with consequent increase in the asymmetrically dimethylated TDG species. Thus, in the presence of high SAM levels, we can argue that PRMT1, deregulated in a wide variety of tumors (Montenegro et al., 2020; Tang et al., 2022), may represent one of the major players ruling dysmetabolism-dependent oncogenic programs, when PRMT5 is inactivated. Several specific PRMT1 inhibitors are currently in clinical trials, supporting the importance of this SAM-dependent enzyme in cancer therapy. The advent of single-cell high-throughput techniques and spatial metabolomics now allows us to gain a comprehensive understanding of the interrelationship between metabolism and transcript profiles in different tumors and even within diverse tumor regions. In parallel, data cross-talk between the metabolome and chromatin methylome may reveal more precisely the link between metabolic alterations and changes in epigenome methylation pattern, indicating potential metabolically vulnerable targets suitable for reverting aberrant epigenomic methylation profiles typical of neoplastic diseases.

## Data Availability

The original contributions presented in the study are included in the article/supplementary material, further inquiries can be directed to the corresponding authors.
